# Supplementary material for: Detecting and understanding meaningful cancerous mutations based on computational models of mRNA splicing
Source: NPJ Syst Biol Appl. 2024 Mar 7;10:25. doi: 10.1038/s41540-024-00351-7 (PMC10920900; doi:10.1038/s41540-024-00351-7)
Supplement: Supplementary file 2 — Supplementray material [file 41540_2024_351_MOESM2_ESM.pdf]

**Supplementary Discussion 1**

Mutations at splice junctions (which disrupt essential GU/AG dinucleotides and necessarily result in a splice site deletion) that cause a change in *SpliceAI* probability of 0.5 or more validate in *RNAseq* at rate *r*, and all other non-splice site mutations causing a probability change above this threshold to validate in RNAseq at $\frac{3}{4}r$ when using this threshold[1]. We chose *SpliceAI* because of its high predictive power and larger context capacity, even compared to other tools such as *MaxEntScan*[2]. *MaxEntScan* was shown to not perform as well in the task of identifying canonical splice sites against *SpliceAI*. Specifically looking at the CFTR gene, *SpliceAI* predicts 26 donors and 26 acceptors, all of which are true, annotated junctions, while MaxEntScan identified 49 acceptors and 22 donors, 9 and 5 of which were true, annotated sites, respectively. The drastic difference in accuracy lends itself in large part to the fact that *MaxEntScan* considers a very small sequence context[2] (9 nucleotides for donors and 23 nucleotides for acceptors against 10K nucleotides in *SpliceAI*) and that *SpliceAI* leverages deep learning, a more sophisticated approach for detecting distant and less important determinants[1]. To further compare the performance, we use VEP to annotate 2,620 SNVs in CFTR with reference and alternate allele *MaxEntScan* scores to check results when handling variants rather than reference gene annotation. First, *MaxEntScan* correlates well with *SpliceAI* in terms of detecting missplicing variants; when classifying a *MaxEntScan*-detected missplicing event as a change in score of 50% relative to the reference allele, we find that out of 36 variants detected as missplicing with *SpliceAI*, 29 are also detected by *MaxEntScan* (p-value: < 1E-5). However, *MaxEntScan* also detects an additional 80 variants, generally agreeing with the previous analysis indicating a high false positive rate with *MaxEntScan*. Moreover, *MaxEntScan* cannot grade missplicing that is caused by mutations outside of the following ranges: (-3 to 6) or (-20, 3) nucleotides from the

donor and acceptor[2]. The high false positive rate and the limited distance of *MaxEntScan* make *SpliceAI* the more suitable tool.

**Supplementary References**


1. Jaganathan, K. *et al.* Predicting Splicing from Primary Sequence with Deep Learning. *Cell* **176**, 535-548.e24 (2019).
2. Yeo, G. & Burge, C. B. Maximum Entropy Modeling of Short Sequence Motifs with Applications to RNA Splicing Signals. *Journal of Computational Biology* **11**, 377–394 (2004).

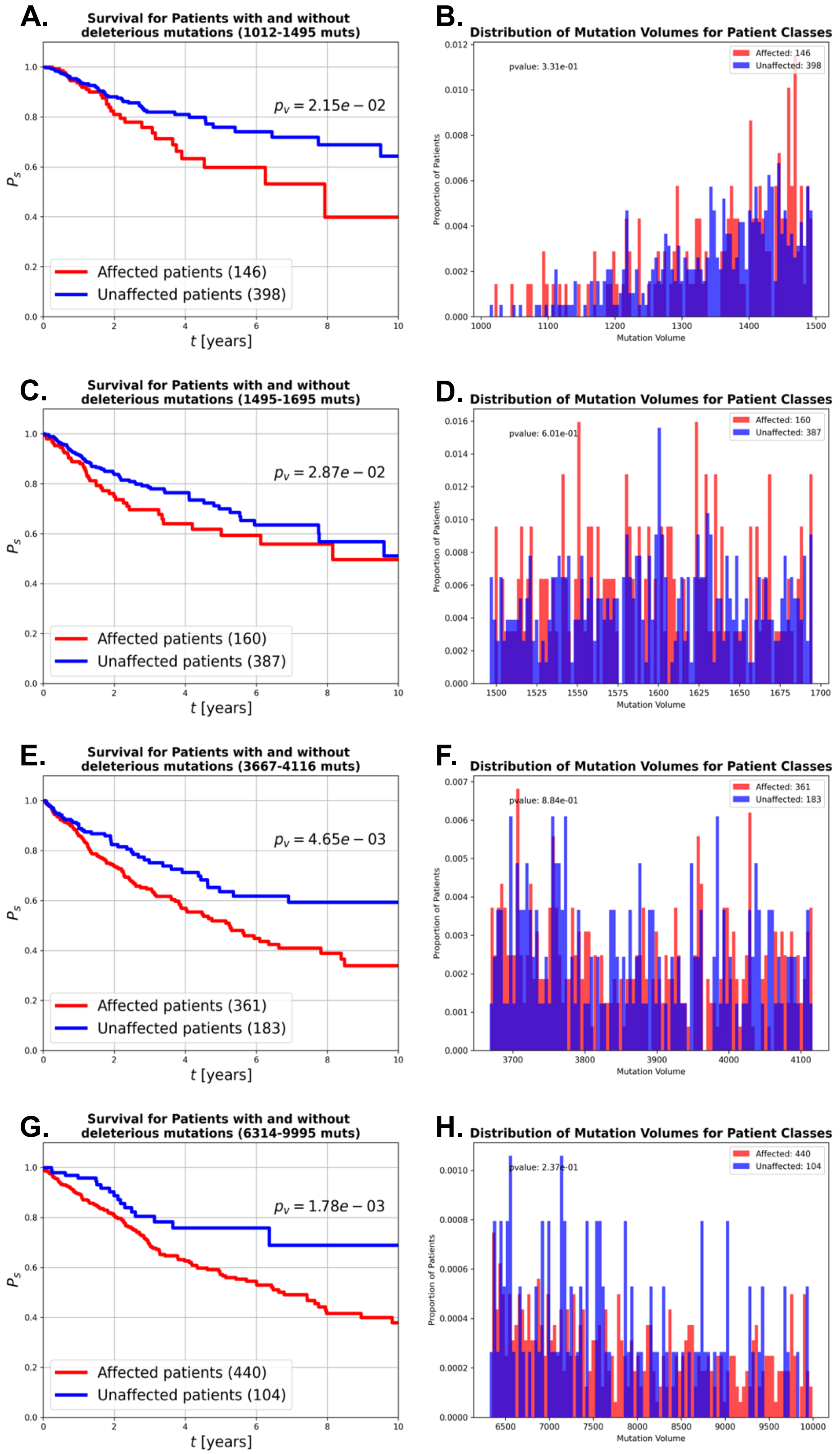


**Supplementary Figure 1 A**. KM survival curves for patients with TMB between 1,012 and 1,495 that carry pathogenic missplicing variants against those that do not. **B.** Mutation count distributions for patients with TMB between 1,012 and 1,495 that carry pathogenic missplicing variants against those that do not. **C.** KM survival curves for patients with TMB between 1,495 and 1,695 that carry pathogenic missplicing variants against those that do not. **D.** Mutation count distributions for patients with TMB between 1,495 and 1,695 that carry pathogenic missplicing variants against those that do not. **E.** KM survival curves for patients with TMB between 3,667 and 4,116 that carry pathogenic missplicing variants against those that do not. **F.** Mutation count distributions for patients with TMB between 3,667 and 4,116 that carry pathogenic missplicing variants against those that do not. **G**. KM survival curves for patients with TMB between 6,314 and 9,995 that carry pathogenic missplicing variants against those that do not. **H.** Mutation count distributions for patients with TMB between 6,314 and 9,995 that carry pathogenic missplicing variants against those that do not.
